# First dose target attainment with extended infusion regimens of piperacillin and meropenem

**DOI:** 10.1186/s13054-025-05445-0

**Published:** 2025-05-22

**Authors:** Gustaf Beijer, Maria Swartling, Elisabet I. Nielsen, Olof Breuer, Christian G. Giske, Erik Eliasson, Johan Petersson

**Affiliations:** 1 MDK, Medical Unit of Clinical Pharmacology, Karolinska University Hospital, 141 86, Huddinge, Stockholm, Sweden; 2 Function Perioperative Medicine and Intensive Care, Karolinska University Hospital, Stockholm, Sweden; 3 MDK, Medical Unit of Clinical Microbiology, Karolinska University Hospital, Stockholm, Sweden; 4 Division of Clinical Pharmacology, Department of Laboratory Medicine, Karolinska Institutet, Stockholm, Sweden; 5 Section of Anaesthesiology & Intensive Care Medicine, Department of Physiology and Pharmacology, Karolinska Institutet, Stockholm, Sweden; 6 Division of Clinical Microbiology, Department of Laboratory Medicine, Karolinska Institutet, Stockholm, Sweden; 7 Department of Pharmacy, Uppsala University, Uppsala, Sweden

**Keywords:** Beta-lactam antibiotics, Intensive care, Therapeutic drug monitoring, Pharmacokinetics, Pharmacodynamics

## Abstract

**Background:**

Standard dosing regimens of meropenem and piperacillin-tazobactam frequently fail to achieve targeted plasma concentrations in critically ill patients. Extended or continuous regimens are often used to improve target attainment. Although prompt antibiotic initiation is a major determinant of survival, few studies have reported systemic concentrations early after treatment initiation. No prior study has reported concentrations immediately after the loading dose and first extended infusion. This study aimed to evaluate plasma target attainment during the first dosing interval with an extended infusion regimen in a general intensive care unit (ICU).

**Methods:**

Adult ICU patients were prospectively included in conjunction with the first administration of meropenem or piperacillin-tazobactam. Treatment was initiated with a 0.5 h loading dose immediately followed by a 3 h extended infusion; typically 4 + 4 g piperacillin or 1(− 2)g + 1(− 2)g meropenem, in line with the local ICU protocol. Patients requiring renal replacement therapy were excluded. Plasma concentrations were measured post-loading dose (C_max_), near the end of the first extended infusion, and at the end of the first dosing interval (C_min_). Samples were analyzed using validated tandem mass spectrometry (UHPLC-MS/MS) methods. The primary endpoint was the proportion of patients achieving 100% time above minimum inhibitory concentrations (*f*T > MIC) during the first dosing interval. This was evaluated using observed C_min_ above 2 mg/L (meropenem) and 20 mg/L (piperacillin). Additionally, published pharmacokinetic models were applied to the observed data for %fT > MIC estimation, using an a posteriori Bayesian approach.

**Results:**

We included 65 meropenem and 142 piperacillin measurements from 22 and 48 patients, respectively. Many patients (45% meropenem, 38% piperacillin) failed to reach 100% *f*T > MIC with the standard regimens used. Target non-attainment was associated with high estimated glomerular filtration rates (eGFR) and suspected augmented renal clearance (ARC). All meropenem patients that failed to reach target had eGFR > 90 mL/min/1.73 m^2^, as did 76% of corresponding piperacillin patients. Patients with suspected ARC frequently exhibited a tenfold or greater peak-to-trough decline (C_min_/C_max_ < 0.1).

**Conclusions:**

Despite aggressive dosing, plasma concentrations often fail to reach 100% *f*T > MIC during the first dosing interval. Alternative regimens and early plasma concentration measurements followed by adaptive dose adjustments should be considered to improve target attainment.

**Supplementary Information:**

The online version contains supplementary material available at 10.1186/s13054-025-05445-0.

## Introduction

The beta-lactam antibiotics piperacillin and meropenem are two of the most common antimicrobials used worldwide against infections in critically ill patients. Like other beta-lactams, their antimicrobial activity is largely time-dependent. [1] This means that effective treatment requires a dosing regimen that achieves concentrations of beta-lactams above the minimum inhibitory concentration (MIC) of relevant microbes for a sufficient (40–70%) part of the dosing interval [2], commonly expressed as e.g. ‘50% *f*T > MIC’, where’T’ denotes time, and ‘*f*’ refers to the free (unbound) drug concentration. More aggressive targets, e.g. 100% *f*T > 1–4 × MIC, are commonly advocated for critically ill patients. [3, 4] However, there is ample evidence that many critically ill patients fail to achieve 100% *f*T > MIC or even 50% *f*T > MIC against worst-case pathogens with standard doses and traditional short infusion regimens of piperacillin and meropenem [5–10]. An important reason for this is the altered pharmacokinetics associated with critical illness [9, 11]. Clinical studies have e.g. demonstrated that larger volumes of distribution and increased renal clearance are common in the critically ill [11]. These observations have contributed to the increasing use of various prolonged infusion regimens in intensive care units [12]. Among prolonged infusion regimens, a distinction is usually made between extended infusions, typically referring to infusion times of 3–4 h, and continuous infusion, respectively.

Clinical studies have shown that early treatment with antibiotics effective against the causative pathogen is a major determinant of survival in critical illness caused by infection [13–16]. To optimize treatment from the start, the goal should be to reach the target *f*T > MIC already after the first antibiotic administration. This critical, early period is, however, largely overlooked in previous studies measuring piperacillin and meropenem concentrations in critically ill patients. 

This study enrolled critically ill patients where treatment with either meropenem or piperacillin was started with an aggressive initial loading dose. The primary aim was to find out how many patients achieved 100% *f*T > MIC during the first dosing interval. We further aimed to characterize the pharmacokinetics of these drugs during the first few hours after the loading dose. Analyses were performed both with and without the support of population pharmacokinetic modelling.

## Materials and methods

### Study design and patient population

This was a single-center, prospective pharmacokinetic study conducted at the adult general intensive care unit (ICU) at Karolinska University Hospital Solna, Sweden. As part of a larger research project, patients were recruited between Nov 2021 and Dec 2023. Patients were eligible for inclusion if they were above 18 years of age and if initiation of piperacillin or meropenem treatment was undertaken in the ICU. Patients who already received the first dose before ICU admission were not eligible, due to the specific study focus on the first dosing interval. Patients who had received the same antibiotic within the previous 96 h were also excluded, as were patients with ongoing or planned renal replacement therapy. Patient enrolment was partly dictated by research nurse availability. The Regional Ethical Review Board in Stockholm approved the study (ref. no. 2017/806-31 and amendment 2018/1658-32). Informed consent to study participation was obtained from patients before blood sampling if possible, or after blood sampling but before data analysis (with assent from the closest relatives) when necessary due to altered consciousness, in accordance with the ethical approval and the Swedish Ethical Review Act (SFS 2003:460).

### Dosing regimens and blood sampling

For both drugs, treatment was started with a loading dose administered over 0.5 h, immediately followed by a 3 h infusion, in accordance with the local ICU protocol. Subsequent doses were administered as 3 h infusions regardless of the dosing interval. The typical piperacillin regimen consisted of a 4 g loading dose (+ 0.5 g tazobactam) followed by a 3 h infusion of 4 g every six (q6h) to eight (q8h) hours, in accordance with national guidelines. For meropenem, loading doses of 1-2 g followed by maintenance infusions of 1–2 g q8h were typically used. For both compounds, prolonged dosing intervals (q12h) were considered in the presence of renal failure. Individual dosing regimens were determined by the treating intensivist with or without input from an infectious disease consultant, as per clinical routine.

Study-related blood sampling for quantification of piperacillin or meropenem in plasma was performed at three time points; immediately after the loading dose (C_max_), during the last 15 min of the first extended (3 h) infusion, and as a trough sample (C_min_) immediately before the second extended infusion. Samples were centrifuged to separate plasma, which was the matrix used for bioanalysis. The study-related concentration measurements did not influence therapeutic decisions.

### Bioanalysis

All samples were analyzed using reversed phase liquid chromatography coupled to tandem mass spectrometry (UHPLC-MS/MS) at the hospital drug laboratory, with accredited methods validated in-house according to European Medicines Agency (EMA) guidelines [17]. The range of quantification was 0.1–150 mg/L for piperacillin and 0.1–100 mg/L for meropenem, respectively. All reported concentrations refer to total, i.e., protein-bound and -unbound, concentrations. In short, the instruments consisted of a Dionex Ultimate 3000RS LC system coupled to a TSQ Quantis triple quadropole mass spectrometer with an electrospray ionization ion source. All parts of the UHPLC-MS/MS system, and the tracefinder software used, were purchased from Thermo Fisher Scientific (Waltham, MA).

### Estimates of renal function

Due to the central role of renal function for the elimination of both compounds, estimated glomerular filtration (eGFR) rates received particular attention in the data analysis. Plasma creatinine (µM) was determined in all patients on the day of study sampling and used as the basis for eGFR and estimated creatinine clearance (CrCL) calculations. Relative eGFR (mL/min/1.73 m^2^) was calculated according to the CKD-EPI2021 formula [18]. Absolute eGFR (mL/min) was calculated from the relative eGFR combined with the Du Bois formula [19] to obtain individual body surface estimates.

Augmented renal clearance (ARC) is common among critically ill patients and typically defined as a relative creatinine clearance (CrCL) > 130 mL/min/1.73 m^2^, preferably based on urinary creatinine measurements [20]. Since we did not measure urinary creatinine in our study, we used estimated absolute CrCL (mL/min) based on plasma creatinine, according to the Cockcroft-Gault Eq. [21] We opted not to convert the calculated CrCL to relative values (mL/min/1.73 m^2^), since dose adjustments are preferably guided by absolute rather than relative clearance estimates. Importantly, estimations from plasma creatinine levels often underestimate creatinine clearance as measured in urine [20], and plasma creatinine can be expected to decrease with a time lag in early phases of ARC. Hence, we defined ‘suspected ARC’ as either an estimated CrCL > 130 mL/min, or a CrCL > 90 mL/min in combination with an elevated ARC score (> 6 points) [22]. The ARC score takes age, trauma history, and the modified sequential organ failure assessment (SOFA) score into account to identify patients at risk for developing ARC, with a proposed cut-off at > 6 points to indicate a high risk of ARC [20, 22]. The ‘suspected ARC’ category in the present study was defined post-hoc, during analysis of collected data and patient characteristics.

Acute kidney injury (AKI) was defined according to the Kidney Disease: Improving Global Outcomes (KDIGO) 2012 clinical practice guidelines [23]. Consequently, an increase in plasma creatinine by ≥ 26.5 µM (≥ 0.3 mg/dL) within 48 h, an increase to ≥ 1.5 times the baseline value within a week, or a low urine output of ≤ 0.5 mL/kg/h for ≥ 6 h were all considered consistent with AKI.

### Pharmacokinetic and pharmacodynamic analyses

The MIC targets used for assessing %*f*T > MIC were based on epidemiological cut-offs for *P. aeruginosa*, as defined by the European Committee on Antimicrobial Susceptibility Testing (EUCAST) [24]. We chose these targets because they correspond to the highest MIC for pathogens considered susceptible to meropenem or piperacillin. Consequently, a target of 2 mg/L was used for meropenem, and 16 mg/L for piperacillin, respectively. [24] Since these targets are based on free (unbound) drug concentrations, our piperacillin total concentration target was 20 mg/L, i.e., corresponding to an assumed plasma protein binding of 20%. This choice was based on manufacturer-reported bindings in the range of 16–30% [25, 26]. For meropenem, protein binding was not considered since it amounts to only a few percent [27] and is typically disregarded.

A trough (C_min_) level of > 2 mg/L (meropenem) and > 20 mg/L (piperacillin) was considered equivalent to achievement of 100% *f*T > MIC for the individual patient. However, C_min_ samples collected in the context of clinical practice are rarely drawn at the exact end of the dosing interval. Furthermore, with the short half-lives of meropenem and piperacillin, even small deviations in sampling time may bias *f*T > MIC estimates when observed C_min_ is collected at a time point other than the exact end of the dosing interval. This is particularly true for borderline C_min_ near the target level. To address this issue, we applied a pharmacokinetic modelling strategy (see below) to estimate individual PK parameters with an a posteriori Bayesian approach. This way, a predicted C_min_ at the exact end of the dosing interval could be determined for each patient. Furthermore, the modeling strategy enabled estimation of %*f*T > MIC, which would have been unattainable with observed concentrations alone. The primary exposure target evaluated was 100% *f*T > MIC, i.e., in line with the target for observed C_min_. A secondary target of 50% *f*T > MIC, and more aggressive targets of 50–100% *f*T > 4xMIC were also evaluated.

Previously published population pharmacokinetic models [28, 29] were applied to the observed data using Monolix version 2021R2 (Lixoft©, Antony, Fr). For meropenem, a two-compartment model with creatinine clearance and age as covariates affecting clearance (CL), and body weight as a covariate affecting the central volume of distribution was used [28]. For piperacillin, a two-compartment model without covariates, developed from critically ill patients was used [29]. These specific models were identified through two external evaluation publications [30, 31] and used after verifying feasible implementation in Monolix. Importantly, some of the models in the external evaluation publications were not applicable to us, e.g. due to inclusion of covariates unavailable in our study population. In some other instances, the less flexible residual error model parameterization in Monolix was likely an issue for some NONMEM-developed models. The final choice of models for the purposes of the present study was guided primarily by visual inspection of individual model fits to our data. Model details, parameter estimates, individual model fits, predictive performance data in our population, and summary statistics of empirical Bayes estimates (EBE) are provided in the Supplementary Appendix.

The models were implemented in Monolix by fixing the initial values of population distribution parameters and variability measures in accordance with the original publications. [28, 29] The mode of the conditional parameter distribution was estimated for every parameter and individual using the Metropolis–Hastings Markov chain Monte Carlo algorithm implemented in Monolix. These individual pharmacokinetic parameter estimates were used to predict individual concentration–time profiles (Supplementary Appendix, Figures S1-S2). Estimates of achieved %*f*T > MIC were obtained with Simulx v.2021R2 (Lixoft©, Antony, Fr), for the duration of the first dosing interval as well as the first 24 h.

Terminal half-life calculations were performed as part of the pharmacokinetic analyses (see Supplementary Appendix for details). However, since half-lives are of limited clinical usefulness for predicting C_min_ after the initial bolus and subsequent 3 h infusion, the actual concentration decrease during the first dosing interval (C_min_/C_max_) was also calculated for every patient. Our interest was to determine whether C_max_ in conjunction with e.g. estimates of renal function might identify patients with a large predicted concentration decrease and therefore at increased risk of C_min_ below target. This could be a useful tool for identifying patients in need of dose adjustments to avoid subtherapeutic concentrations at the end of the dosing interval. In the calculations of C_min_/C_max_ ratios, observed rather than model-predicted C_min_ and C_max_ were used except in the subset of piperacillin patients with dosing intervals of eight or 12 h, for whom model-predicted concentrations six hours after the start of the extended infusion were used as C_min_. In the few cases where the loading dose amount differed from the maintenance dose amount, the C_min_ and C_max_ were dose normalized to standard meropenem (1 g) and piperacillin (4 g) doses before calculating the C_min_/C_max_ ratio.

### Statistical analyses

R version 4.4.0 was used for descriptive statistics and statistical analyses including half-life calculations, Wilcoxon rank sum exact tests (to compare group differences in C_min_, half-lives, and C_min_/C_max_ ratios), receiver operating characteristic (ROC) and cumulative distribution analyses (CDA). Summary statistics are reported as median and interquartile range (IQR) for non-normally distributed data if not otherwise specified.

## Results

A total of 142 piperacillin samples from 48 patients and 65 meropenem samples from 22 patients were collected. Patient characteristics are presented in Table 1. Most patients (n = 48/70; 69%) were treated for hospital-acquired pneumonia (HAP) including ventilator-associated pneumonia (VAP). The majority of patients displayed a normal or increased eGFR with a median value of 102 (IQR: 79–115) mL/min/1.73 m^2^. Estimated CrCL consistent with ARC was seen in 59% (n = 13) of patients treated with meropenem and 38% (n = 18) of patients treated with piperacillin (Table 1). The corresponding proportions of patients showing ‘suspected’ ARC were 68% (n = 15) and 40% (n = 19). Conversely, acute kidney injury was seen in 32% (n = 7) and 17% (n = 8) of patients, respectively.

**Table 1:** Patient characteristics, clinical and laboratory data

	Meropenem (n = 22)	Piperacillin (n = 48)
Age (years)	58 (46–69)	63 (46–70)
Male sex	16 (73%)	35 (73%)
Height (cm)	178 (175–180)	175 (168–182)
Weight (kg)	83 (68–94)	85 (72–101)
BMI (kg/m^2^)	27 (23–30)	27 (23–31)
Plasma albumin (g/L)	29 (22–30)	27 (25–32)
Plasma creatinine (µmol/L)	58 (46–78)	74 (59–94)
Relative eGFR (mL/min/1.73 m^2^)	106 (92–117)	95 (76–112)
Absolute eGFR (mL/min)	123 (98–130)	113 (89–126)
CrCL (mL/min)	144 (99–176)	112 (70–147)
Augmented renal clearance	13 (59%)	18 (38%)
Acute kidney injury during ICU stay	7 (32%)	8 (17%)
*Cause of ICU admission*		
Medical	12 (55%)	40 (83%)
Trauma	9 (41%)	8 (17%)
Elective surgery	1 (4%)	0 (0%)
Hospital-acquired infection	18 (82%)	36 (75%)
*Verified or suspected focus of infection*		
Lower respiratory tract	15 (68%)	45 (94%)
Other (urinary, abdominal, CNS)	7 (32%)	3 (6%)
Pathogen isolated (yes)	11 (50%)	33 (69%)
Gram-negative (and mix)	8 (73%)	21 (64%)
Gram-positive	3 (27%)	12 (36%)
Invasive ventilation	17 (77%)	38 (79%)
Vasopressor therapy	17 (77%)	37 (77%)
SAPS3 score	64 (60–75)	64 (52–74)
SOFA score	8 (6–10)	8 (7–10)
Mortality 30 days after ICU admission	7 (32%)	4 (8%)
ICU mortality*	6 (27%)	1 (2%)

Continuous variables are presented as median (IQR). Categorical variables presented as count (%). Measures of renal function, SOFA score, vasopressor therapy, and invasive ventilation status refer to the day of study sampling. SAPS3 scores were recorded upon ICU admission. Pathogen information refers to culture results that were considered representative of the causative pathogen. ARC, augmented renal clearance; BMI, body mass index; CrCL, creatinine clearance calculated according to the Cockroft-Gault formula. eGFR = estimated glomerular filtration rate. SAPS3, simplified acute physiology score 3; SOFA, sequential organ failure

*All ICU deaths occurred within 30 days of admission

## Meropenem

### Observations

The administered loading dose was 1 g in 14 (64%) of the patients, 2 g in six patients (27%), and 0.5 g in two patients (Table 2). The subsequent first extended infusion was 1 g for 12 (55%) of the patients, and 2 g for the remaining ten. The intended dosing interval was 8 h in all patients.

**Table 2:** Exposure and target attainment based on observed and model-predicted data

	Meropenem (n = 22)		Piperacillin (n = 48)	
	Observed	Predicted	Observed	Predicted
Cmin (mg/L)	2.7 (0.2–28)	2.6 (0.2–27)	29 (1.9–130)	31 (4.2–127)
*f*Cmin/MIC	1.3 (0.1–14)	1.3 (0.1–14)	1.5 (0.1–6.5)	1.5 (0.2–6.3)
No. (%) of patients not reaching target (100% *f*T > MIC)	9 (41%)	10 (45%)	17 (35%)	18 (38%)
*Achieved exposure in patients not reaching target*				
% *f*T > MIC (first DI)		78 (57–99)		79 (63–98)
% *f*T > MIC (first 24 h)		74 (54–97)		76 (58–93)
Actual dosing regimen (no. of patients; %)	1 g + 1 g q8h	(n = 11; 50%)	4 g + 4 g q6h	(n = 34; 71%)
	2 g + 2 g q8h	(n = 6; 27%)	4 g + 4 g q8h	(n = 9; 19%)
	1 g + 2 g q8h	(n = 3; 14%)	Other**	(n = 5; 10%)
	Other*	(n = 2; 9%)		

^*^ = 0.5 g + 1-2 g q8h

** = 2-4 g + 2-4 g q6h-q12h. DI = Dosing interval. qXh = every X hours. MIC = Minimum inhibitory concentration, here referring to the targets of 2 mg/L (meropenem) and 20 mg/L (piperacillin), respectively. N/A = Not applicable. Values are presented as median (range) if not otherwise specified. Observed values refer to actually measured plasma concentrations. Predicted values refer to model-predicted concentrations

**Fig. 1:**
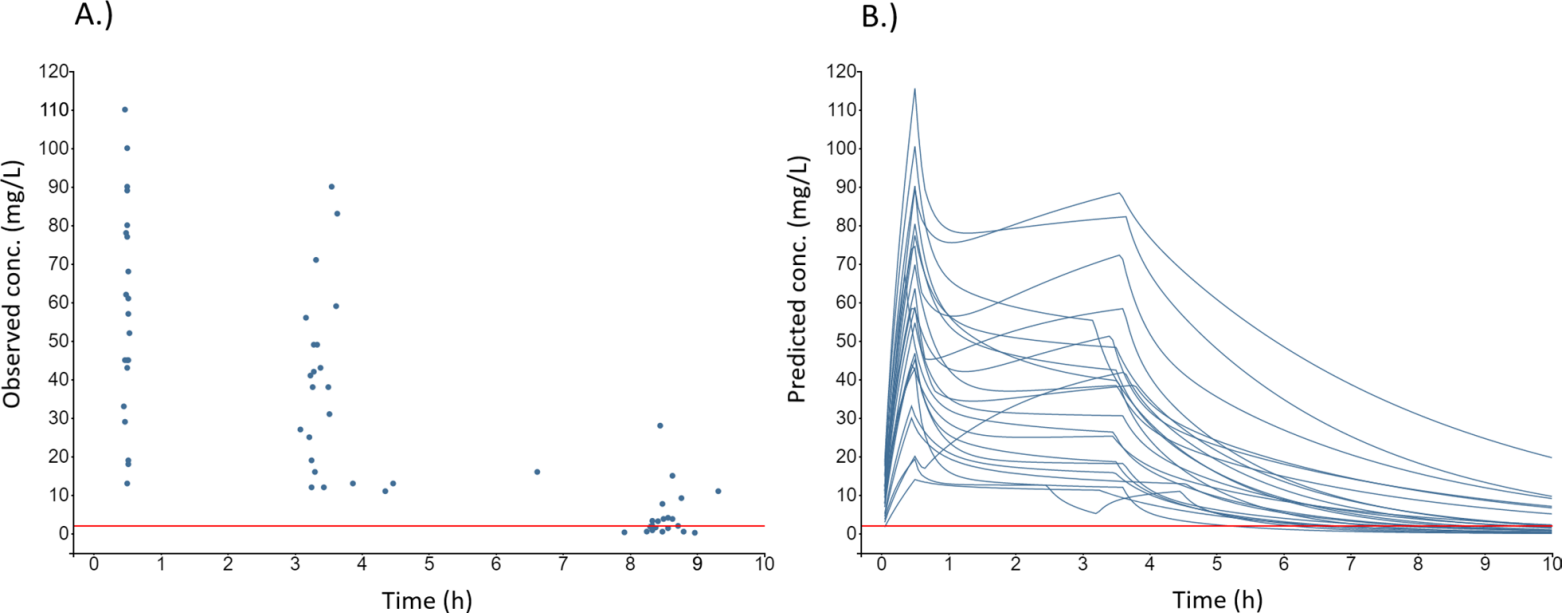
Individual meropenem observations and model predictions. Observed plasma concentrations of meropenem (n = 65) versus time after start of the loading dose (**A**), and model-predicted concentration–time profiles for each individual (n = 22) (**B**). The horizontal red line corresponds to the targeted Cmin threshold of 2 mg/L. Conc. = Concentration

All observed meropenem concentrations are presented in Fig. 1A. The observed median C_min_ was 2.7 (IQR: 1.0–6.8, range: 0.2–28) mg/L. Of the 22 meropenem patients, nine (41%) failed to reach the primary target of 100% *f*T > MIC based on an observed C_min_ < 2.0 mg/L after the first extended infusion. In one patient, the C_min_ sample was collected almost two hours (1.88 h) earlier than planned. All other C_min_ samples were collected within ± 0.75 h from the end of the planned dosing interval, 82% (n = 18/22) within ± 0.5 h, and 73% within ± 0.25 h.

The majority of patients with observed C_min_ < 2.0 mg/L (n = 7/9) received a 1 g loading dose followed by a 1 g extended infusion. Among the six patients receiving the highest 2 g + 2 g regimen (Table 2), only one had an observed Cmin < 2 mg/L. However, an additional two had borderline observed Cmin of 2.0 and 2.1 mg/L, respectively.

### Model-based predictions

The predicted individual concentration–time profiles according to the *Li *et al. model [28] are presented in Fig. 1B.

Of the 22 patients, ten (45%) failed to reach the primary target of 100% *f*T > MIC according to the model-based predictions. Nine of these ten patients also had an observed Cmin < 2.0 mg/L, whereas one of them had an observed Cmin of 2.1 mg/L. In this case, the Cmin blood sample was drawn 10 min before the end of the 8 h dosing interval. The model-predicted concentration for this patient at the actual sampling time was 2.1 mg/L (i.e. same as the observed concentration), which fell to a model-predicted 1.9 mg/L by the end of the dosing interval. All patients reached > 50% *f*T > MIC (Table 2), and 73% (n = 16/22) also reached > 50% *f*T > 4xMIC. Five patients (23%) reached 100% *f*T > 4xMIC according to the model predictions. Four of these five patients met the criteria for AKI before treatment initiation.

Among patients with a predicted exposure below 100% *f*T > MIC, the median predicted *f*T > MIC was 78% (Table 2). The corresponding median predicted time spent below MIC was 1.5 h (range 0.1–3.4 h).

The dosing regimen was 1 g q8h in eight of the ten patients failing to reach 100% *f*T > MIC. One of these patients received a reduced loading dose of 0.5 g meropenem. The two remaining patients failed to achieve 100% *f*T > MIC despite receiving a 2 g loading dose followed by a 2 g extended infusion.

The mortality after 30 days among meropenem patients was 32% (n = 7/22). Mortality was 33% among patients that reached target (n = 4/12) and 30% (n = 3/10) among patients failing to reach the target.

## Piperacillin

### Observations

The administered loading dose was 4 g in 44 (92%) of the patients, 2 g in three patients, and 3 g in one patient. The subsequent first extended infusion was 4 g in 45 (94%) of the patients, 2 g in two patients, and 3 g in one patient. The planned dosing interval was 6 h in 38 (79%) of the patients, 8 h in nine (19%) of the patients, and 12 h in one patient.

**Fig. 2:**
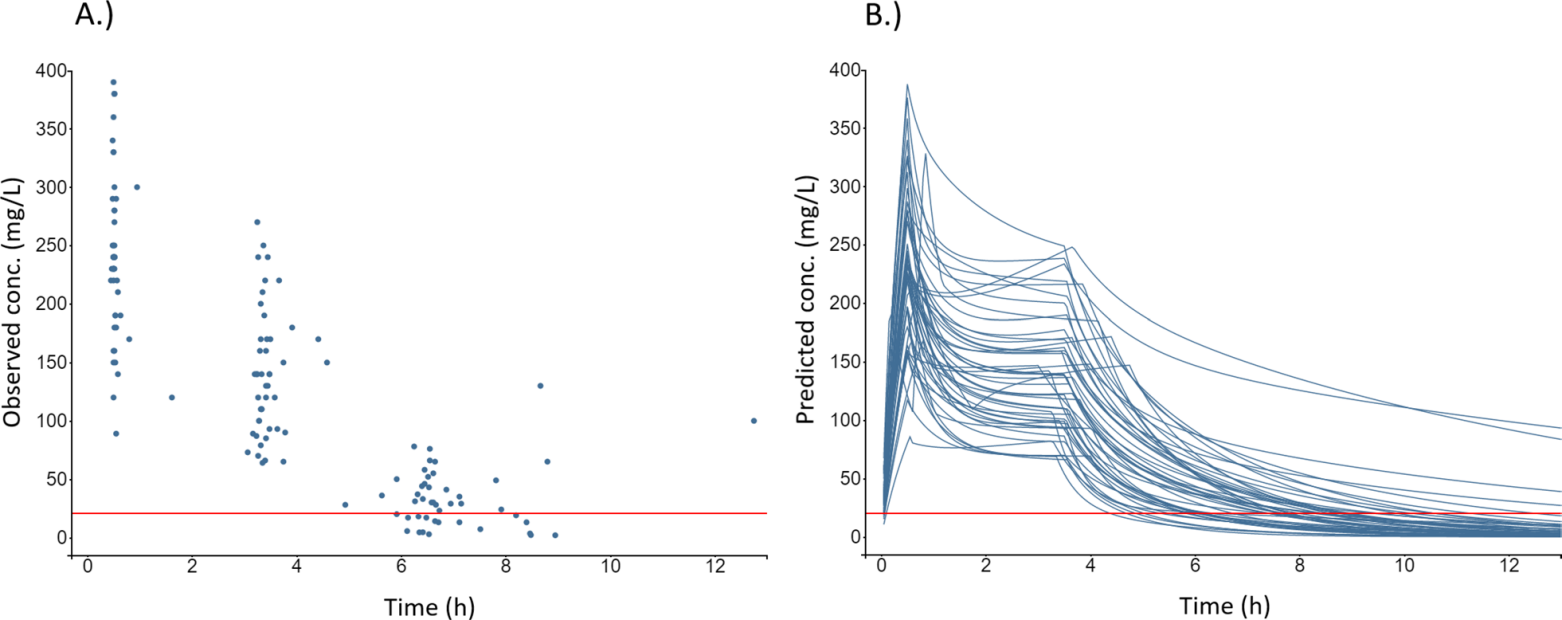
Individual piperacillin observations and model predictions. Observed plasma concentrations of piperacillin (n = 142) versus time after start of the loading dose (**A**), and model-predicted concentration–time profiles for each patient (n = 48) (**B**). The horizontal red line corresponds to the targeted Cmin threshold of 20 mg/L. Conc. = Concentration

All observed concentrations are presented in Fig. 2A. The median observed C_min_ was 29 (IQR: 14–48, range: 1.9–130) mg/L. Of the 48 piperacillin patients, 17 (35%) failed to reach the primary target of 100% *f*T > MIC based on an observed C_min_ < 20 mg/L after the first extended infusion. The C_min_ sampling times were generally in good line with protocol, with a median deviation of less than five minutes (− 0.08 h) from the end of the planned dosing interval. In one patient, the C_min_ sample was drawn 1.75 h earlier than planned (4.25 h after extended infusion start). All other C_min_ samples were drawn within ± 1.0 h from the end of the planned dosing interval, 83% (n = 40/48) within ± 0.5 h, and 75% (n = 36/48) within ± 0.3 h (± 19 min).

The majority of patients with observed C_min_ < 20 mg/L (n = 16/17) received a 4 g loading dose followed by a 4 g extended infusion.

### Model-based predictions

The *Fillâtre *et al*.* model [29], when applied to our observed data, predicted individual concentration–time profiles in accordance with Fig. 2B.

Of the 48 patients, 18 (38%) failed to reach 100% *f*T > MIC according to the model-based predictions. Sixteen of these 18 patients also had an observed C_min_ < 20 mg/L, whereas two of the patients had observed C_min_ of 20 mg/L and 28 mg/L, respectively. These Cmin samples were both mistimed (collected 42 min and 105 min before the end of the dosing interval, respectively), which explained the model-predicted C_min_ < 20 mg/L at the end of the dosing intervals. In one case, a difference between observed and model-predicted target attainment was not due to mistimed sampling, but rather a poor individual model fit to the observed data (Supplementary appendix, Fig. S1A&B, subject “P43”).

All patients reached a model-predicted > 50% *f*T > MIC (Table 2), the majority of whom (n = 35/48, 73%) also reached > 50% *f*T > 4xMIC. Two patients (4%) reached 100% *f*T > 4xMIC, both of whom displayed AKI according to KDIGO [23] criteria.

Among the 18 patients with *f*T > MIC below 100%, the median predicted *f*T > MIC was 79% (Table 2) and the corresponding median time spent below target was 1.3 h (range 0.1–3.0 h). In 13 (72%) of these patients, the dosing interval was q6h, with the remaining five patients (28%) receiving piperacillin q8h. One of the five patients failing to reach 100% *f*T > MIC with q8h dosing would have reached the target with q6h dosing, according to the model-based predictions.

The mortality after 30 days among piperacillin patients was 8% (n = 4/48). Mortality was 10% among patients that reached target (n = 3/30), and 6% (n = 1/18) among patients failing to reach the target.

### C_min_ in relation to renal function

**Fig. 3:**
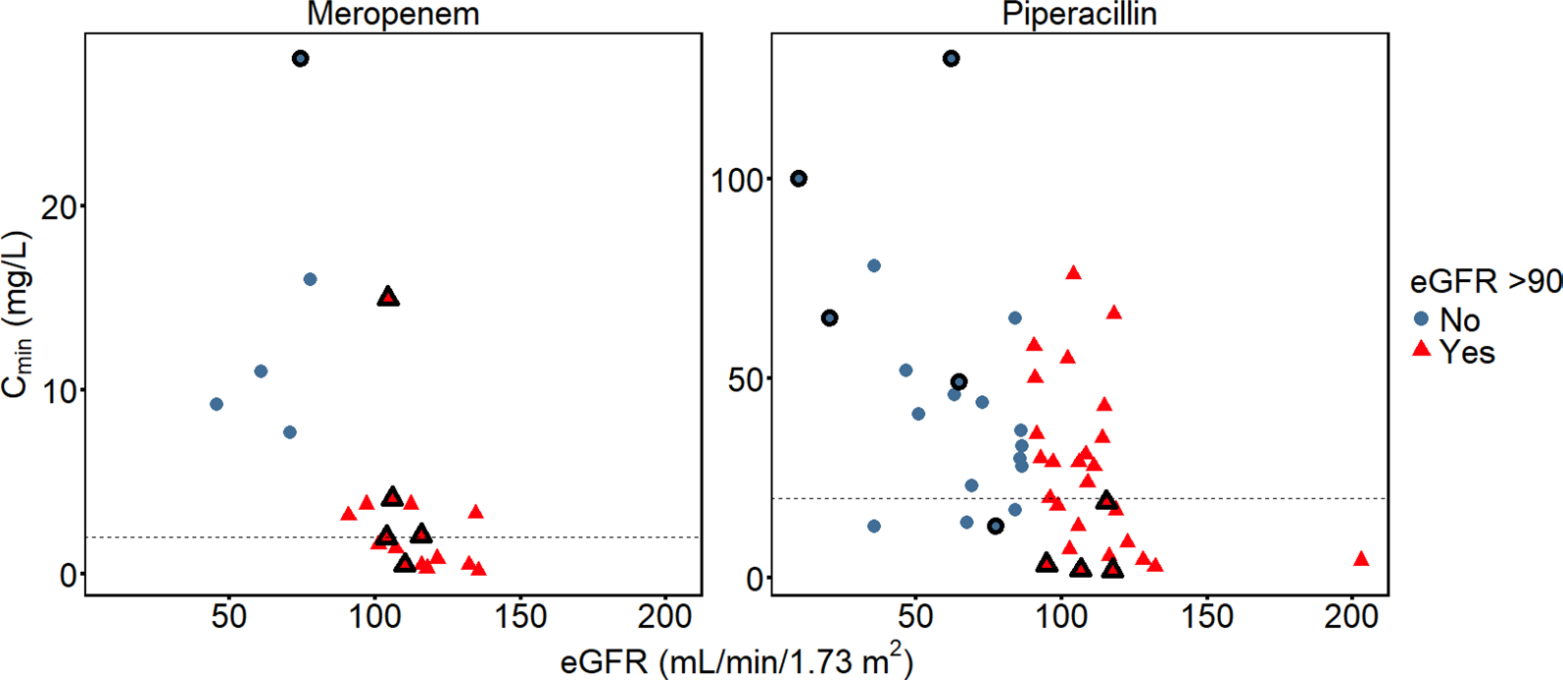
Trough concentrations versus estimated glomerular filtration rates. Observed trough plasma concentrations (Cmin) of meropenem and piperacillin in relation to eGFR. Each data point represents a single patient. Individuals represented by red triangles all displayed an eGFR > 90 mL/min/1.73 m^2^ on the day of study sampling. Blue circles correspond to eGFR ≤ 90 mL/min/1.73 m^2^. The dosing interval was 8 h for all meropenem patients, and the most common loading dose amount was 1 g (n = 14/22; 64%) followed by a 1 g extended infusion (n = 12/22; 55%). Patients receiving the highest doses of 2 g + 2 g meropenem are highlighted with black borders, for comparison. For piperacillin, the most common loading dose amount was 4 g (n = 44/48; 92%) followed by a 4 g extended infusion (n = 45/48; 94%), and the most common dosing interval was 6 h (n = 38/48; 79%). Piperacillin patients with longer dosing intervals (8 h or 12 h) are highlighted with black borders. eGFR = estimated glomerular filtration rate according to the CKD-EPI2021 formula

Estimated GFR showed a clear inverse correlation with Cmin levels. As seen in Fig. 3, all meropenem patients with a sub-target C_min_ had an eGFR > 90 mL/min/1.73m^2^, as did 76% (n = 13/17) of corresponding piperacillin patients. Conversely, among patients with eGFR > 90 mL/min/1.73 m^2^, 59% (n = 10/17) and 50% (n = 14/28) failed to reach the C_min_ target for meropenem and piperacillin, respectively.

The median C_min_ in patients with eGFR > 90 mL/min/1.73m^2^ was 1.9 mg/L for meropenem and 22 mg/L for piperacillin. In contrast, median C_min_ in patients with eGFR < 90 mL/min/1.73 m^2^ was 11 mg/L and 39 mg/L, respectively (Fig. 3). These C_min_ differences between eGFR groups were statistically significant (meropenem difference 95% CI 6.0–16, P = 0.002, and piperacillin difference 95% CI 5.0–32, P = 0.01, respectively).

All nine meropenem patients that failed to reach the C_min_ target (Fig. 3) met our criteria for suspected ARC, of whom seven (78%) met standard ARC criteria. Among the 17 piperacillin patients failing to reach the C_min_ target, 65% met both criteria for ARC.

### Predictive potential of early samples

Peak concentrations (C_max_) of meropenem after the loading dose ranged between 13 and 110 mg/L (Figs. 1A&B), with a median C_max_ of 57 (IQR: 43–78) mg/L. The corresponding C_max_ range for piperacillin was 89–390 mg/L (Figs. 2A&B) with a median of 230 (IQR: 190–280) mg/L.

Increased GFR estimates were generally associated with a more rapid decrease in serum concentration. The median terminal half-life of meropenem among patients with eGFR > 90 mL/min/1.73 m^2^ was 1.4 h (IQR: 1.2–1.5 h), which could be compared with 2.7 h (IQR: 2.6–3.0 h) among patients with eGFR < 90 mL/min/1.73 m^2^. Corresponding median half-lives for piperacillin patients were 1.6 h (IQR: 1.3–2.4 h) and 2.2 h (IQR: 1.8–3.2 h). For both drugs, these differences between eGFR groups were statistically significant (*p* = 0.001, and *p* = 0.008, respectively).

**Fig. 4:**
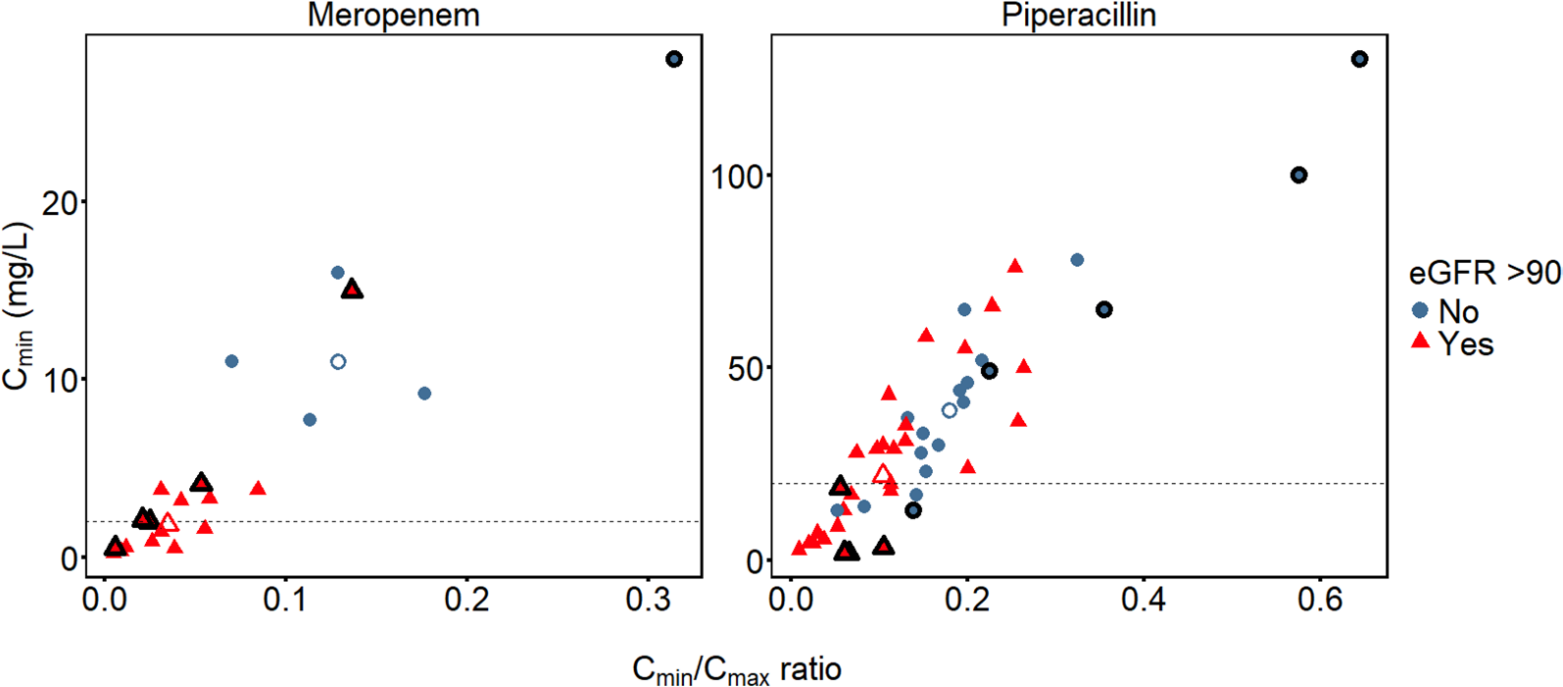
Trough concentrations versus Cmin/Cmax ratio. Observed trough concentrations (Cmin) in relation to the ratio between Cmin and Cmax for every patient. The Cmin/Cmax ratio was dose-normalized for six patients (n = 5 meropenem, n = 1 piperacillin) that received differing loading dose and maintenance dose amounts. Furthermore, observed Cmin was substituted with model-predicted 6 h concentration when calculating the Cmin/Cmax ratio in piperacillin patients with longer (> 6 h) dosing intervals of 8 h (n = 9) or 12 h (n = 1). Empty triangles represent median values for patients with an eGFR > 90 mL/min/1.73 m^2^. Empty circles represent median values for patients with eGFR ≤ 90 mL/min/1.73 m^2^. Observations highlighed with black borders correspond to patients receiving the highest meropenem doses (2 g loading dose + 2 g extended infusion), or the longest (8–12 h) piperacillin dosing intervals, respectively. Dashed lines represent the Cmin targets of 2 mg/L (meropenem) and 20 mg/L (piperacillin). eGFR = estimated glomerular filtration rate according to the CKD-EPI2021 formula

In Fig. 4, the relationship between the C_min_/C_max_ ratio and C_min_ is shown for both drugs. The median (range) C_min_/C_max_ ratio for meropenem patients with eGFR > 90 mL/min/1.73 m^2^ was 0.03 (< 0.01–0.14), compared with 0.13 (0.07–0.31) in patients with eGFR < 90 mL/min/1.73 m^2^. For piperacillin patients, the corresponding median (range) C_min_/C_max_ ratio was 0.1 (< 0.01–0.26) in patients with eGFR > 90 mL/min/1.73 m^2^, and 0.19 (0.05–0.65) in patients with eGFR < 90 mL/min/1.73 m^2^. For both drugs, the ratio differences between groups were statistically significant (meropenem difference 95% CI 0.05–0.18, *P* = 0.003, and piperacillin difference 95% CI 0.03–0.13, *P* = 0.002, respectively).

**Fig. 5:**
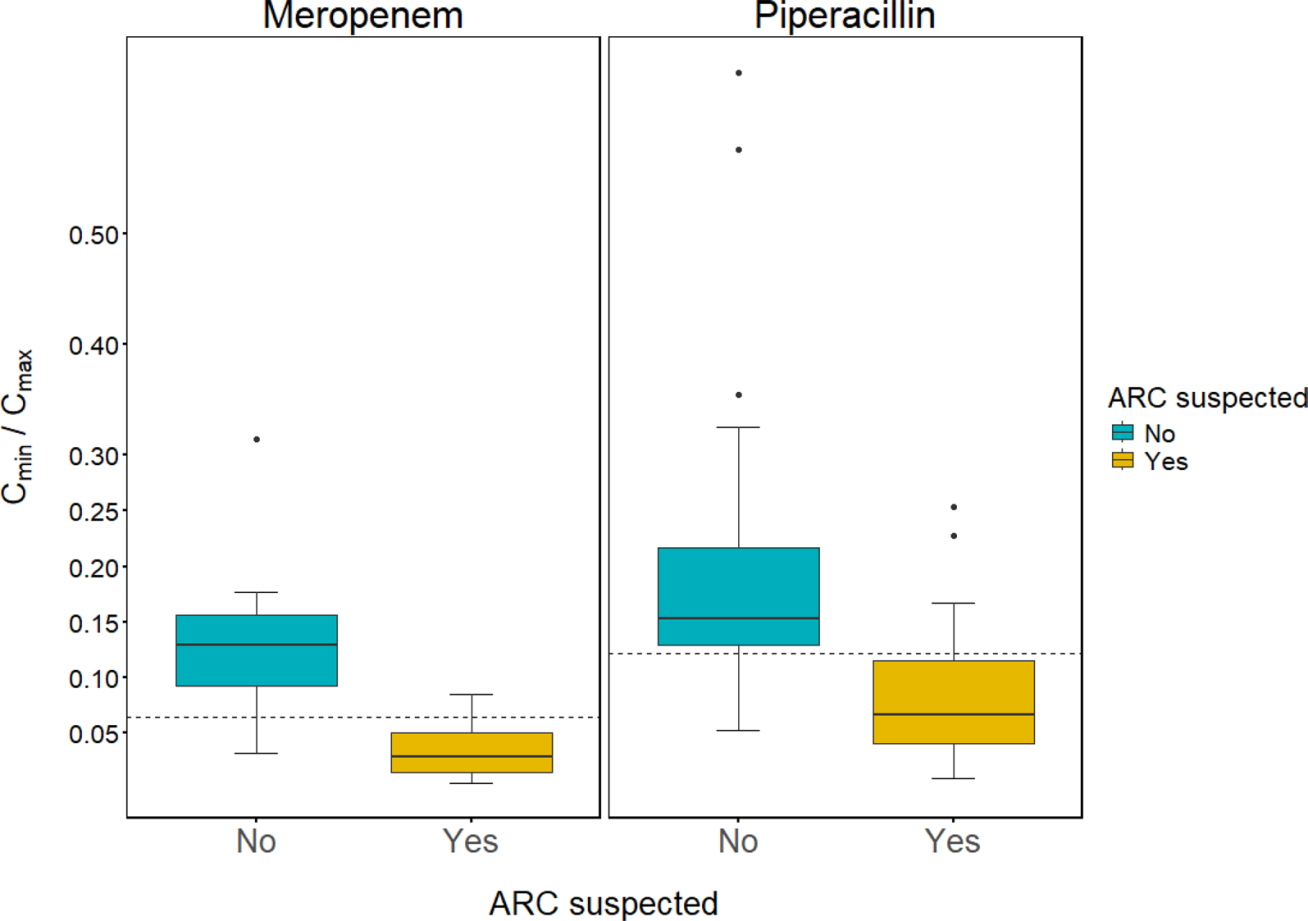
Cmin/Cmax ratio in patients with and without suspected ARC. Boxplot demonstrating the observed ratios between trough (Cmin) and peak (Cmax) concentrations in patients with and without suspected ARC. The Cmin/Cmax ratio was dose-normalized for six patients (n = 5 meropenem, n = 1 piperacillin) that received differing loading dose and maintenance dose amounts. Furthermore, observed Cmin was substituted with model-predicted 6 h concentrations in the subset of piperacillin patients with longer (> 6 h) dosing intervals of 8 h (n = 9) or 12 h (n = 1). Yellow boxes represent patients with a CrCL > 130 mL/min on the day of study sampling, or an ARC score > 6 in combination with a CrCL > 90 mL/min. Dashed lines indicate optimal cut-offs (meropenem 0.06, piperacillin 0.12) according to receiver operating characteristic (ROC) analysis. ARC = Augmented renal clearance. CrCL = estimated creatinine clearance (mL/min)

All meropenem patients with suspected augmented renal clearance showed a C_min_/C_max_ ratio < 0.1, i.e., a greater than tenfold reduction of plasma concentrations during the first dosing interval, as is evident from Fig. 5. As also seen in this figure, this was true for the majority (56%) of piperacillin patients as well, although a slightly higher cut-off to differentiate between ARC and non-ARC patients was suggested. Receiver operating characteristic (ROC) and cumulative distribution analysis (CDA) suggested C_min_/C_max_ ratio cut-offs of approximately 0.06 for meropenem and 0.12 for piperacillin based on ARC status in our study population. These cut-offs are indicated with dashed lines in Fig. 5. ROC curves and CDA plots are provided in the Supplementary appendix (Figures S3, S4).

## Discussion

To the best of our knowledge, this is the first study to report actual target attainment during the critical first dosing interval after initiation of treatment with an extended infusion regimen of piperacillin or meropenem in critically ill patients. Our results demonstrate that at the start of treatment, similar dosing regimens result in plasma concentrations that differ widely between patients. More importantly, despite aggressive dosing a large percentage of patients fail to reach 100% *f*T > MIC, a target that is commonly recommended for critically ill patients [3, 4].

In similarity with many other investigators, we used target concentrations corresponding to worst-case scenario pathogens. Since infections are often caused by bacteria with a lower MIC, higher actual *f*T > MIC against causative pathogens can generally be expected [32]. Nevertheless, for most infected critically ill patients the causative pathogen is not verified. We believe that, at least at the start of treatment, it is reasonable to aim for 100% *f*T > MIC against all relevant pathogens intended to be covered. For critically ill patients, this certainly includes *P. aeruginosa*, especially in the context of hospital-acquired infections.

### Dosing regimens

Clinical pharmacokinetic studies have previously demonstrated the superiority of extended versus short infusion regimens in terms of PK/PD target attainment but also noted that even extended infusion regimens fail to achieve 100% *f*T > MIC in many critically ill patients based on sampling after > 24 h of therapy [33, 34]. Our study adds to this body of evidence that sub-target exposures are common even during the first dosing interval after the loading dose, i.e., after a total of 2 g of meropenem or 8 g of piperacillin administered during 6–8 h. Although the importance of the loading dose and its exact timing when initiating extended infusion regimens is currently unclear [2], it makes theoretical sense to strive for achievement of therapeutic concentrations as quickly as possible. Judging from the high observed C_max_ levels achieved in the present study, administering at least part of the loading dose as an extended rather than a short infusion could arguably contribute more effectively to reaching the *f*T > MIC target. However, loading dose practices during beta-lactam therapy represents a general area in need of further investigation [2].

### Importance of renal function

Estimated or measured creatinine clearance, or other renal function proxies, are frequently identified as significant covariates describing piperacillin and meropenem clearance in population pharmacokinetic models developed from critically ill patients [35, 36]. This is not surprising due to the important role of renal function for the elimination of both compounds. A correlation between measured creatinine clearance and target non-attainment has also been reported for both short and extended infusions of meropenem and piperacillin [8, 33]. With mean half-lives of approximately 1 h for both drugs at normal elimination capacity, no significant increase in C_min_ concentrations between dosing intervals is expected with continued treatment. Under such conditions, the highest concentrations and longest *f*T > MIC are expected during the first dosing interval which, unlike subsequent dosing intervals, is initiated with a loading dose. With this in mind, our results are particularly unsettling.

Patients with a low eGFR (< 30–60 mL/min) were poorly represented in our study, which limits any conclusions to be drawn for patients with renal impairment. However, the fact that six out of the seven patients in total that achieved the more aggressive target of 100% *f*T > 4 X MIC against worst-case pathogens showed acute kidney injury was interesting, and reinforces the notion that this is probably an overly aggressive target for non-continuous regimens [37].

### Augmented renal clearance

Augmented renal clearance (ARC) has been reported to occur in 20–65% of intensive care patients with incidence varying with age and reason for ICU admission [20]. Similar to our results, several prior studies have shown that ARC is associated with an increased incidence of meropenem and piperacillin concentrations below target [8, 20, 33, 38, 39]. Alternative dosing regimens with extended or continuous infusions have thus been suggested among strategies to mitigate the effects of ARC on achieved beta-lactam concentrations. Importantly, our results show that even after a loading dose immediately followed by an extended infusion, many ARC patients still fail to reach the targeted plasma concentrations. These findings suggest that a more individualized dosing approach is warranted to better account for patients with verified or suspected ARC.

### ***The potential value of C***_***max***_*** sampling***

An increased interest in C_max_ sampling early during beta-lactam therapy could enable faster identification of patients at risk of subtherapeutic exposure. In hospitals with short turn-around-times (< 3−4 h) of therapeutic drug monitoring (TDM), a sample drawn during the first hour can be analyzed and reported during the first extended infusion interval. Under such circumstances, dose adjustments can be made without delay to prevent low C_min_ from the outset.

The preferable approach to predict the C_min_ based on an observed C_max_ would be to use model-informed dosing, based on a validated population model with relevant covariates. However, this requires advanced software and efficient communication between bedside physicians and pharmacokinetic modellers, where the latter may not be immediately available at all times. The relatively simple C_min_/C_max_ ratio concept, as a measure of the approximate peak-to-trough concentration decline to expect with a standardized regimen, could be of value as a complementary tool.

As a practical example, prolonging the planned infusion time from 3 h to 4-5 h, or starting the next extended infusion immediately upon receiving a piperacillin C_max_ result of 200 mg/L in a young trauma patient with ventilator-associated pneumonia and CrCL > 90 mL/min is appealing from a risk–benefit perspective. According to the findings of the present study, such a patient might run a > 50% risk of experiencing a tenfold or greater reduction in plasma concentration during the dosing interval (C_min_/C_max_ ratio < 0.1, see Fig. 5), which would translate into a sub-target C_min_ of < 20 mg/L. Since the toxicity risk is not expected to increase with a prolonged infusion duration, the case for such an approach would seem strong. Even in the presence of substantial GFR overestimation, the downside of prolonging the infusion duration is probably limited based on the range of observed C_min_/C_max_ ratios (< 0.01–0.65) in our study population. The accuracy of C_min_ predictions can probably be increased by adding clinical judgment to e.g., distrust unreasonable eGFR estimates, and considering other predictors including established ARC risk factors. Special attention to documentation quality is also important [40], to ensure that correct dosing and sampling information is obtained.

There are practical clinical advantages of considering net peak-to-trough declines, rather than e.g. half-lives, when assessing an obtained C_max_ with the standardized regimens in focus of this study, where a 0.5 h loading dose is immediately followed by a 3 h extended infusion. Here, the net concentration declines reflect a complex interplay of post-loading distribution, clearance, and ongoing drug input, not captured by half-lives alone.

Importantly, the C_min_/C_max_ ratio is specific to the studied regimen. Dose-normalization can accommodate the use of slightly differing dose amounts, but not significant alterations of infusion times.

### Strengths and limitations

One limitation of our study was the number of enrolled patients and the comparatively few patients with community-acquired infections, which reflects the case-mix at our unit. The use of plasma creatinine-based estimates of GFR instead of measured creatinine clearance was another limitation, that also hampered the precision of ARC identification. The small number of included patients with poor renal function is important to consider, and the fact that we did not measure concentrations of the beta-lactamase inhibitor tazobactam. Also, the reliance on total rather than unbound piperacillin concentration measurements, with an assumption of 20% protein binding in all patients, was a limitation, in addition to the high MIC assumptions corresponding to worst-case scenario pathogens.

Our results should be interpreted in relation to the aggressive initial dosing used in our centre, with the immediate start of a 3 h extended infusion after administering a 0.5 h loading dose. Deferring the first extended infusion slightly, by starting it 1–2 h after the loading dose, has been suggested [7] and would probably increase *f*T > MIC during the first dosing interval. A higher *f*T > MIC would also be expected with longer-duration (e.g. 4 h) extended infusion regimens used in some centres. Future studies should address the potential impact of varying loading dose practices and different extended infusion times on PK/PD target attainment and clinical outcomes in relation to continuous infusion. 

## Conclusions

Despite aggressive dosing with a loading dose immediately followed by a 3 h extended infusion, plasma concentrations often fail to reach 100% *f*T > MIC during the first dosing interval in ICU patients treated with meropenem or piperacillin. Consequently, alternative dosing regimens might be warranted, especially in patients with increased renal clearance. Estimates of GFR and early measurements of antibiotic plasma concentrations after the first administration might be complementary strategies to improve target attainment.

## Supplementary Information

Supplementary file 1

## Data Availability

The data that support the findings of the study are available from the authors upon reasonable request.

## Abbreviations

AKI: Acute kidney injury
ARC: Augmented renal clearance
C_max_: Maximum concentration
C_min_: Minimum concentration
CrCL: Creatinine clearance
eGFR: Estimated glomerular filtration rate
EMA: European Medicines Agency
EUCAST: European Committee on Antimicrobial Susceptibility Testing
*f*: Free (unbound)
HAP: Hospital-acquired pneumonia
ICU: Intensive care unit
MIC: Minimum inhibitory concentration
PK/PD: Pharmacokinetic/pharmacodynamic
T: Time
TDM: Therapeutic drug monitoring
UHPLC-MS/MS: Ultra-high performance liquid chromatography-tandem mass spectrometry
VAP: Ventilator-associated pneumonia

## References

[1] Craig WA. Pharmacokinetic/pharmacodynamic parameters: rationale for antibacterial dosing of mice and men. Clin Infect Dis. 1998;26(1):1–10. 9455502 10.1086/516284

[2] Hong LT, Downes KJ, FakhriRavari A, Abdul-Mutakabbir JC, Kuti JL, Jorgensen S, et al. International consensus recommendations for the use of prolonged-infusion beta-lactam antibiotics: endorsed by the American College of Clinical Pharmacy, British Society for Antimicrobial Chemotherapy, Cystic Fibrosis Foundation, European Society of Clinical Microbiology and Infectious Diseases, Infectious Diseases Society of America, Society of Critical Care Medicine, and Society of Infectious Diseases Pharmacists. Pharmacotherapy. 2023;43(8):740–77. 37615245 10.1002/phar.2842

[3] Abdul-Aziz MH, Alffenaar JC, Bassetti M, Bracht H, Dimopoulos G, Marriott D, et al. Antimicrobial therapeutic drug monitoring in critically ill adult patients: a Position Paper. Intensive Care Med. 2020;46(6):1127–53. 32383061 10.1007/s00134-020-06050-1 PMC7223855

[4] Guilhaumou R, Benaboud S, Bennis Y, Dahyot-Fizelier C, Dailly E, Gandia P, et al. Optimization of the treatment with beta-lactam antibiotics in critically ill patients-guidelines from the French Society of Pharmacology and Therapeutics (Société Française de Pharmacologie et Thérapeutique-SFPT) and the French Society of Anaesthesia and Intensive Care Medicine (Société Française d’Anesthésie et Réanimation-SFAR). Crit Care. 2019;23(1):104. 30925922 10.1186/s13054-019-2378-9 PMC6441232

[5] Varghese JM, Roberts JA, Lipman J. Antimicrobial pharmacokinetic and pharmacodynamic issues in the critically ill with severe sepsis and septic shock. Crit Care Clin. 2011;27(1):19–34. 21144984 10.1016/j.ccc.2010.09.006

[6] Roberts JA, Paul SK, Akova M, Bassetti M, De Waele JJ, Dimopoulos G, et al. DALI: defining antibiotic levels in intensive care unit patients: are current β-lactam antibiotic doses sufficient for critically ill patients? Clin Infect Dis. 2014;58(8):1072–83. 24429437 10.1093/cid/ciu027

[7] Tilanus A, Drusano G. Optimizing the use of beta-lactam antibiotics in clinical practice: a test of time. Open Forum Infect Dis. 2023;10(7):ofad305. 37416756 10.1093/ofid/ofad305 PMC10319623

[8] Petersson J, Giske CG, Eliasson E. Standard dosing of piperacillin and meropenem fail to achieve adequate plasma concentrations in ICU patients. Acta Anaesthesiol Scand. 2016;60(10):1425–36. 27655029 10.1111/aas.12808

[9] Taccone FS, Laterre PF, Dugernier T, Spapen H, Delattre I, Wittebole X, et al. Insufficient β-lactam concentrations in the early phase of severe sepsis and septic shock. Crit Care. 2010;14(4):R126. 20594297 10.1186/cc9091 PMC2945087

[10] Smekal AK, Furebring M, Eliasson E, Lipcsey M. Low attainment to PK/PD-targets for β-lactams in a multi-center study on the first 72 h of treatment in ICU patients. Sci Rep. 2022;12(1):21891. 36535989 10.1038/s41598-022-25967-9 PMC9763385

[11] Gonçalves-Pereira J, Póvoa P. Antibiotics in critically ill patients: a systematic review of the pharmacokinetics of β-lactams. Crit Care. 2011;15(5):R206. 21914174 10.1186/cc10441 PMC3334750

[12] Williams PG, Tabah A, Cotta MO, Sandaradura I, Kanji S, Scheetz MH, et al. International survey of antibiotic dosing and monitoring in adult intensive care units. Crit Care. 2023;27(1):241. 37331935 10.1186/s13054-023-04527-1 PMC10278304

[13] Kumar A, Roberts D, Wood KE, Light B, Parrillo JE, Sharma S, et al. Duration of hypotension before initiation of effective antimicrobial therapy is the critical determinant of survival in human septic shock. Crit Care Med. 2006;34(6):1589–96. 16625125 10.1097/01.CCM.0000217961.75225.E9

[14] Kumar A, Ellis P, Arabi Y, Roberts D, Light B, Parrillo JE, et al. Initiation of inappropriate antimicrobial therapy results in a fivefold reduction of survival in human septic shock. Chest. 2009;136(5):1237–48. 19696123 10.1378/chest.09-0087

[15] Ferrer R, Martin-Loeches I, Phillips G, Osborn TM, Townsend S, Dellinger RP, et al. Empiric antibiotic treatment reduces mortality in severe sepsis and septic shock from the first hour: results from a guideline-based performance improvement program. Crit Care Med. 2014;42(8):1749–55. 24717459 10.1097/CCM.0000000000000330

[16] Kollef MH, Sherman G, Ward S, Fraser VJ. Inadequate antimicrobial treatment of infections: a risk factor for hospital mortality among critically ill patients. Chest. 1999;115(2):462–74. 10027448 10.1378/chest.115.2.462

[17] European Medicines Agency. ICH guideline M10 on bioanalytical method validation and study sample analysis. (2022)

[18] Inker LA, Eneanya ND, Coresh J, Tighiouart H, Wang D, Sang Y, et al. New creatinine- and cystatin C-based equations to estimate GFR without race. N Engl J Med. 2021;385(19):1737–49. 34554658 10.1056/NEJMoa2102953 PMC8822996

[19] du Bois D. Clinical calorimetry: tenth paper a formula to estimate the approximate surface area if height and weight be known. Arch Intern Med. 1916;XVII(6_2):863. 10.1001/archinte.1916.00080130010002.

[20] Bilbao-Meseguer I, Rodríguez-Gascón A, Barrasa H, Isla A, Solinís M. Augmented renal clearance in critically ill patients: a systematic review. Clin Pharmacokinet. 2018;57(9):1107–21. 29441476 10.1007/s40262-018-0636-7

[21] Cockcroft DW, Gault MH. Prediction of creatinine clearance from serum creatinine. Nephron. 1976;16(1):31–41. 1244564 10.1159/000180580

[22] Udy AA, Roberts JA, Shorr AF, Boots RJ, Lipman J. Augmented renal clearance in septic and traumatized patients with normal plasma creatinine concentrations: identifying at-risk patients. Crit Care. 2013;17(1):R35. 23448570 10.1186/cc12544 PMC4056783

[23] Khwaja A. KDIGO clinical practice guidelines for acute kidney injury. Nephron Clin Pract. 2012;120(4):c179–84. 22890468 10.1159/000339789

[24] EUCAST. Antimicrobial wild type distributions of microorganisms. Epidemiological cut-off values (ECOFF) and tentative epidemiological cut-off values (TECOFF). Available via www.eucast.org. [Internet]. [Cited 2024-09-13]

[25] Hospira Healthcare Corp. Product monograph - Piperacillin for Injection (Piperacillin Sodium), 2018. Available from https://pdf.hres.ca/dpd_pm/00044150.PDF. Accessed Sep 2024. [Internet]. 2018

[26] Wockhardt UK Ltd. Piperacillin/Tazobactam 4g/0.5g Powder for Solution for Infusion (SmPC), 2023. Available at: https://www.medicines.org.uk/emc/product/6526/smpc#gref, Cited 13 Sep 2024. [Internet]

[27] Summary of product characteristics. Meropenem STADA. 1000 mg powder for solution for injection or infusion. [Available from: https://www.lakemedelsverket.se/sv/sok-lakemedelsfakta/lakemedel/20110405000094/meropenem-stada-500-mg-pulver-till-injektions-infusionsvatska-losning

[28] Li C, Kuti JL, Nightingale CH, Nicolau DP. Population pharmacokinetic analysis and dosing regimen optimization of meropenem in adult patients. J Clin Pharmacol. 2006;46(10):1171–8. 16988206 10.1177/0091270006291035

[29] Fillâtre P, Lemaitre F, Nesseler N, Schmidt M, Besset S, Launey Y, et al. Impact of extracorporeal membrane oxygenation (ECMO) support on piperacillin exposure in septic patients: a case-control study. J Antimicrob Chemother. 2021;76(5):1242–9. 33569597 10.1093/jac/dkab031

[30] Yang N, Wang J, Xie Y, Ding J, Wu C, Liu J, et al. External evaluation of population pharmacokinetic models to inform precision dosing of meropenem in critically ill patients. Front Pharmacol. 2022;13:838205. 35662716 10.3389/fphar.2022.838205 PMC9157771

[31] Greppmair S, Brinkmann A, Roehr A, Frey O, Hagel S, Dorn C, et al. Towards model-informed precision dosing of piperacillin: multicenter systematic external evaluation of pharmacokinetic models in critically ill adults with a focus on Bayesian forecasting. Intensive Care Med. 2023;49(8):966–76. 37439872 10.1007/s00134-023-07154-0 PMC10425489

[32] Smekal AK, Furebring M, Lipcsey M, Giske CG. Swedish multicentre study of target attainments with β-lactams in the ICU: Which MIC parameter should be used? J Antimicrob Chemother. 2023;78(12):2895–901. 37897332 10.1093/jac/dkad327 PMC10689903

[33] Carlier M, Carrette S, Roberts JA, Stove V, Verstraete A, Hoste E, et al. Meropenem and piperacillin/tazobactam prescribing in critically ill patients: Does augmented renal clearance affect pharmacokinetic/pharmacodynamic target attainment when extended infusions are used? Crit Care. 2013;17(3):R84. 23642005 10.1186/cc12705 PMC4056350

[34] De Waele J, Carlier M, Hoste E, Depuydt P, Decruyenaere J, Wallis SC, et al. Extended versus bolus infusion of meropenem and piperacillin: a pharmacokinetic analysis. Minerva Anestesiol. 2014;80(12):1302–9. 24762706

[35] El-Haffaf I, Caissy JA, Marsot A. Piperacillin-tazobactam in intensive care units: a review of population pharmacokinetic analyses. Clin Pharmacokinet. 2021;60(7):855–75. 33876381 10.1007/s40262-021-01013-1

[36] Dhaese SAM, Farkas A, Colin P, Lipman J, Stove V, Verstraete AG, et al. Population pharmacokinetics and evaluation of the predictive performance of pharmacokinetic models in critically ill patients receiving continuous infusion meropenem: a comparison of eight pharmacokinetic models. J Antimicrob Chemother. 2019;74(2):432–41. 30376103 10.1093/jac/dky434

[37] Dilworth TJ, Schulz LT, Micek ST, Kollef MH, Rose WE. β-lactam therapeutic drug monitoring in critically ill patients: weighing the challenges and opportunities to assess clinical value. Crit Care Explor. 2022;4(7):e0726. 35815181 10.1097/CCE.0000000000000726 PMC9259115

[38] Udy AA, Varghese JM, Altukroni M, Briscoe S, McWhinney BC, Ungerer JP, et al. Subtherapeutic initial beta-lactam concentrations in select critically ill patients: association between augmented renal clearance and low trough drug concentrations. Chest. 2012;142(1):30–9. 22194591 10.1378/chest.11-1671

[39] Huttner A, Von Dach E, Renzoni A, Huttner BD, Affaticati M, Pagani L, et al. Augmented renal clearance, low β-lactam concentrations and clinical outcomes in the critically ill: an observational prospective cohort study. Int J Antimicrob Agents. 2015;45(4):385–92. 25656151 10.1016/j.ijantimicag.2014.12.017

[40] Swartling M, Tängdén T, Lipcsey M, Jönsson S, Nielsen EI. Therapeutic drug monitoring of vancomycin and meropenem: illustration of the impact of inaccurate information in dose administration time. Int J Antimicrob Agents. 2024;63(1):107032. 37956952 10.1016/j.ijantimicag.2023.107032

