# Supplementary material for: First dose target attainment with extended infusion regimens of piperacillin and meropenem
Source: Crit Care. 2025 May 22;29:208. doi: 10.1186/s13054-025-05445-0 (PMC12096718; doi:10.1186/s13054-025-05445-0)
Supplement: Supplementary file 1 [file 13054_2025_5445_MOESM1_ESM.docx]

**Supplementary Appendix**

**
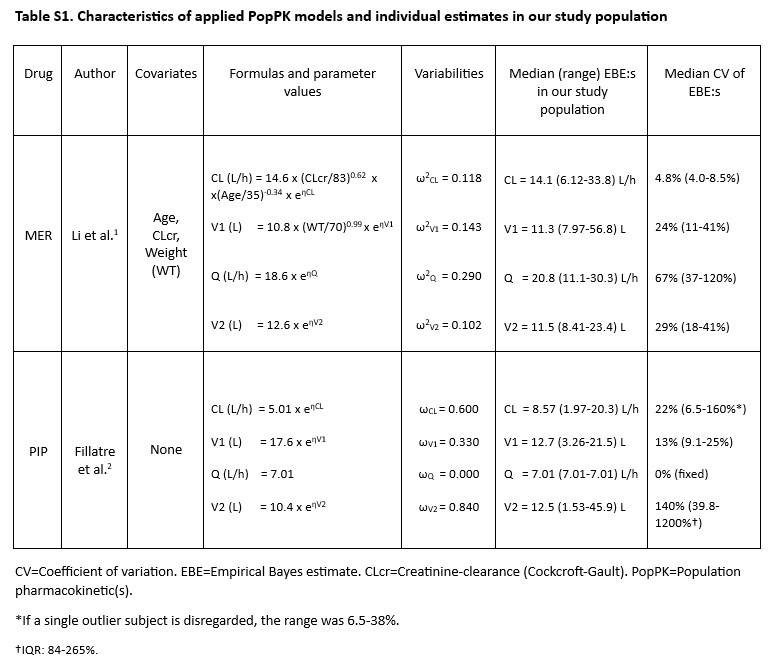
Table S1. Characteristics of applied PopPK models and individual estimates in our study population**


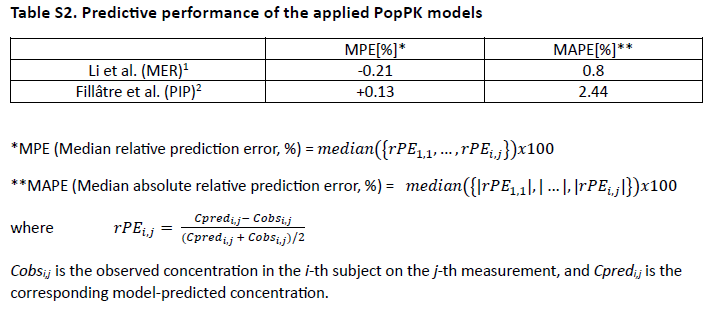
**Table S2. Predictive performance of the applied PopPK models**

**Individual model fits**


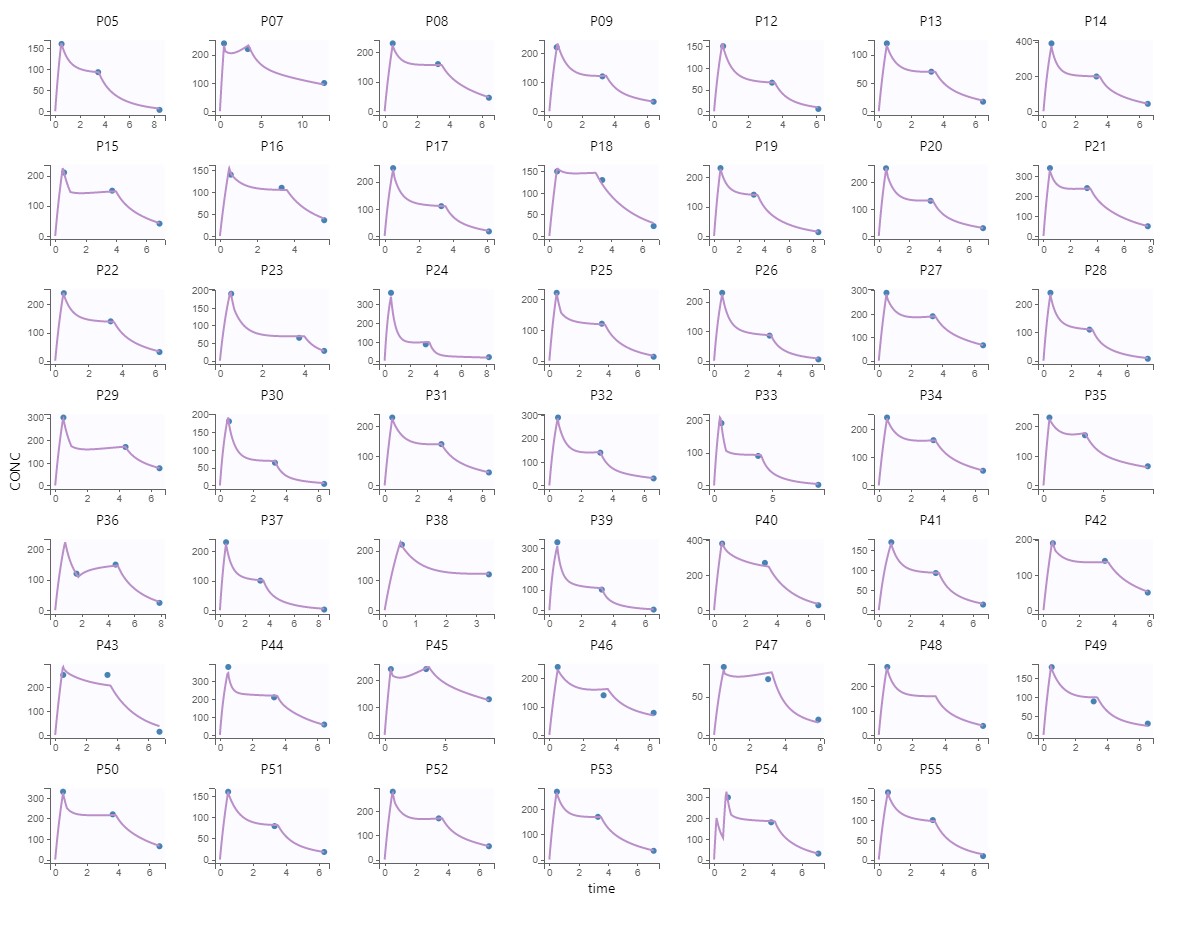
Figure S1A. Individual fits to the *Fillâtre et al.* model (PIP). Individual axis limits.


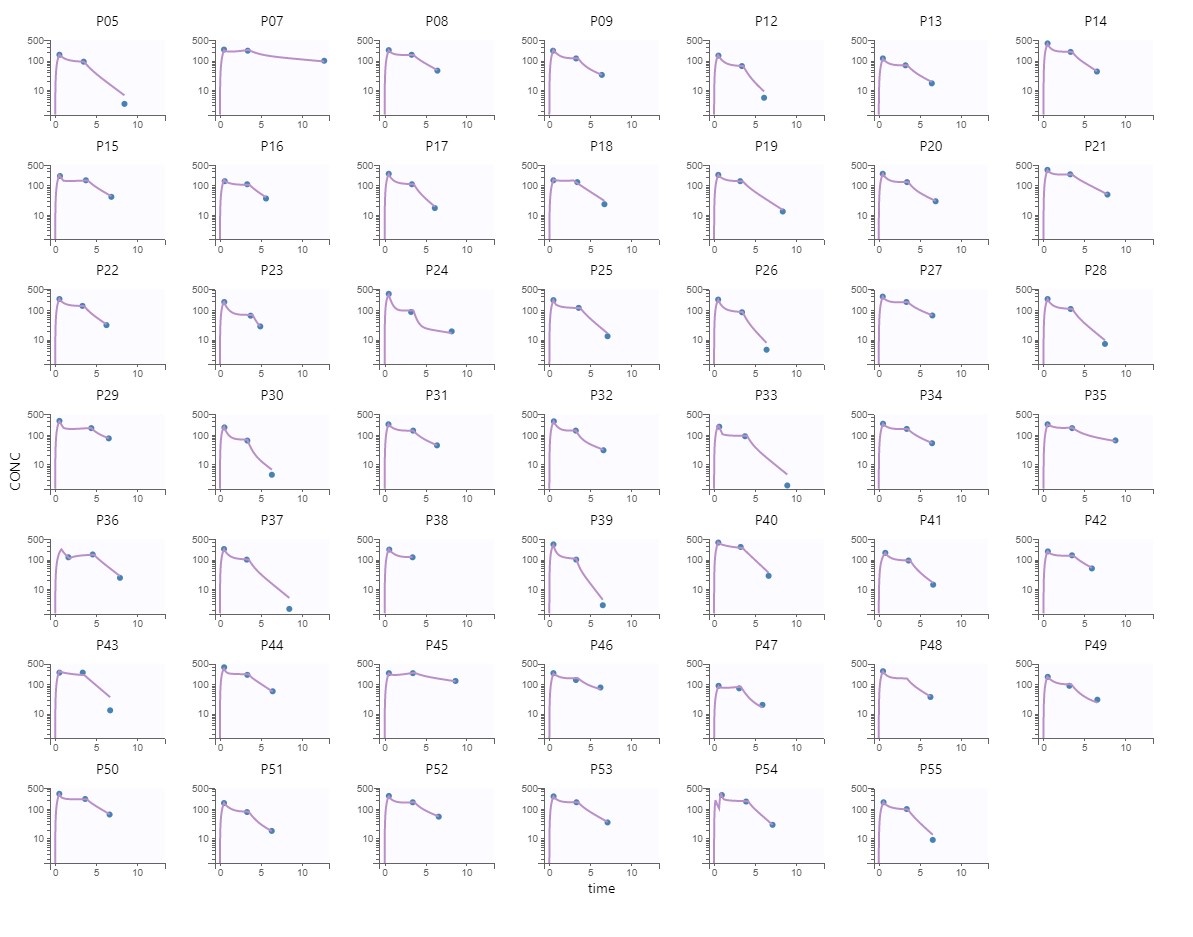
Figure S1B. Individual fits to the *Fillâtre et al.* model (PIP). LogConc, same axis limits.


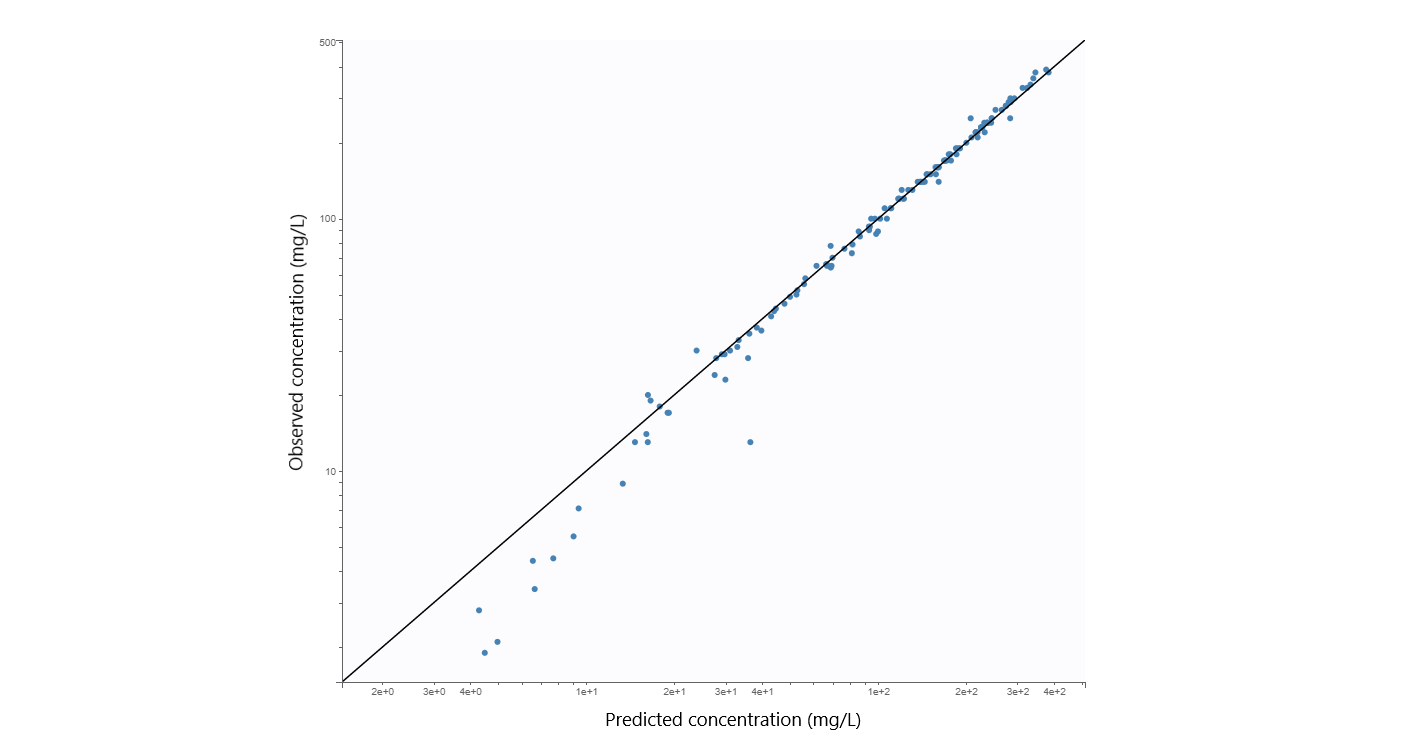
Figure S1C. Individual observations vs model predictions (*Fillâtre et al.* model, PIP)


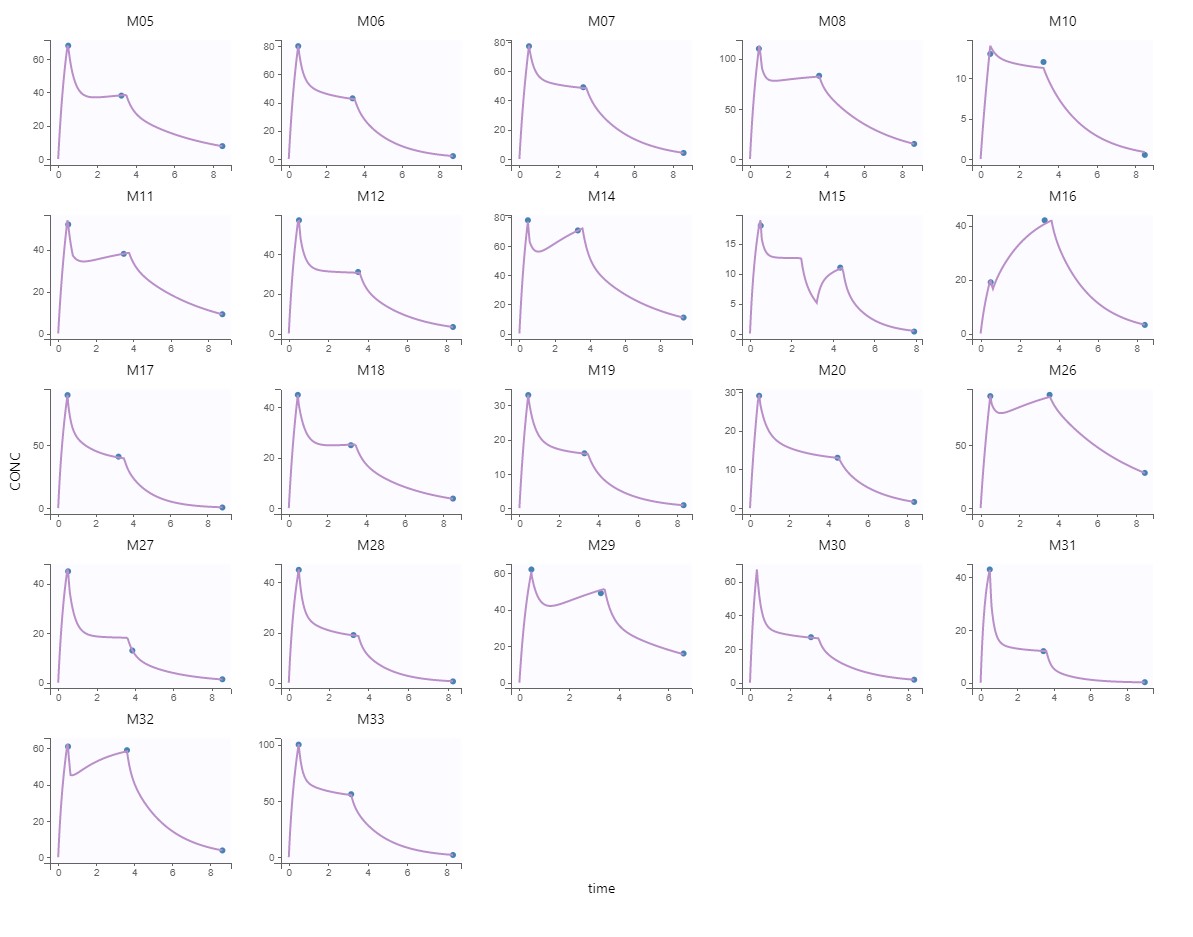
Figure S2A. Individual fits to the *Li et al.* model (MER). Individual axis limits.


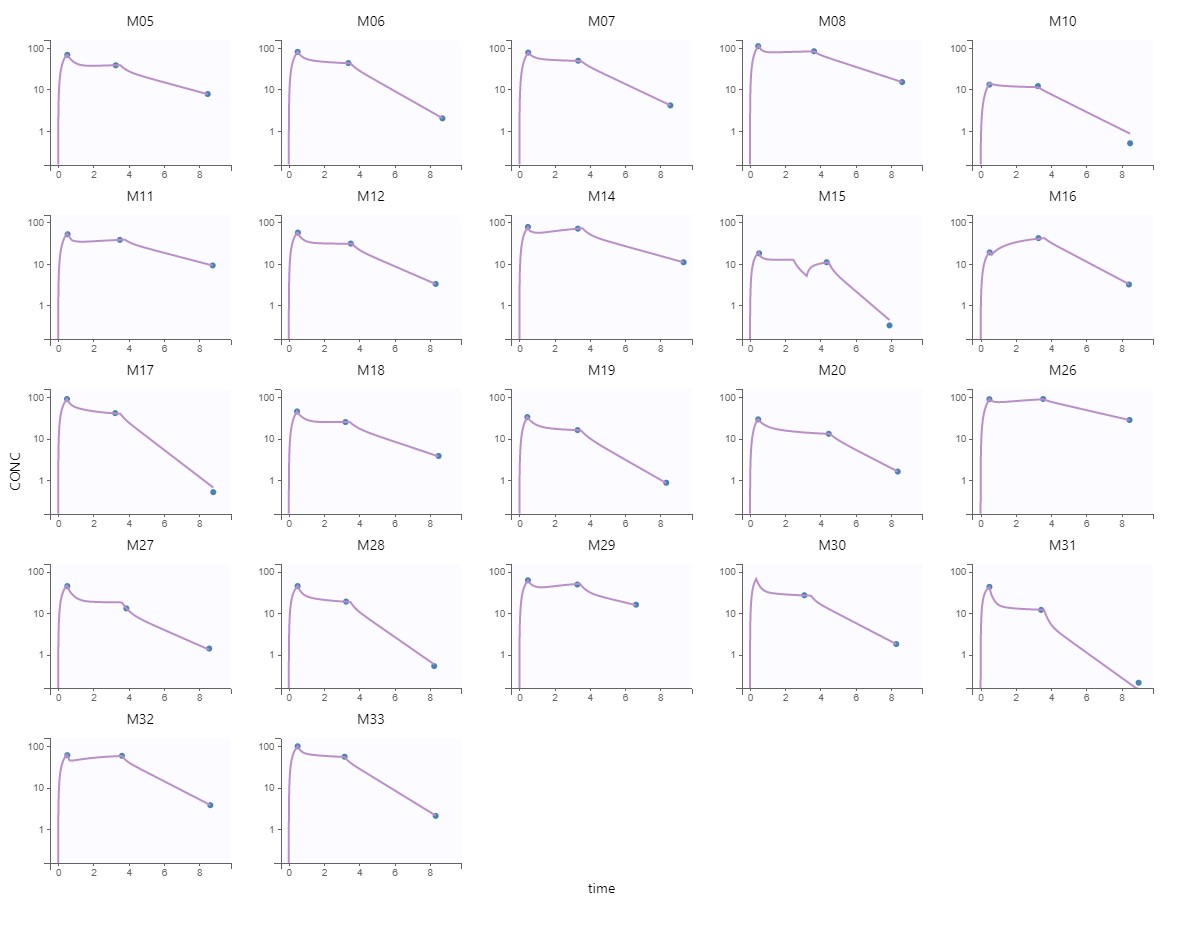
Figure S2B. Individual fits to the *Li et al.* model (MER). LogConc, same axis limits.


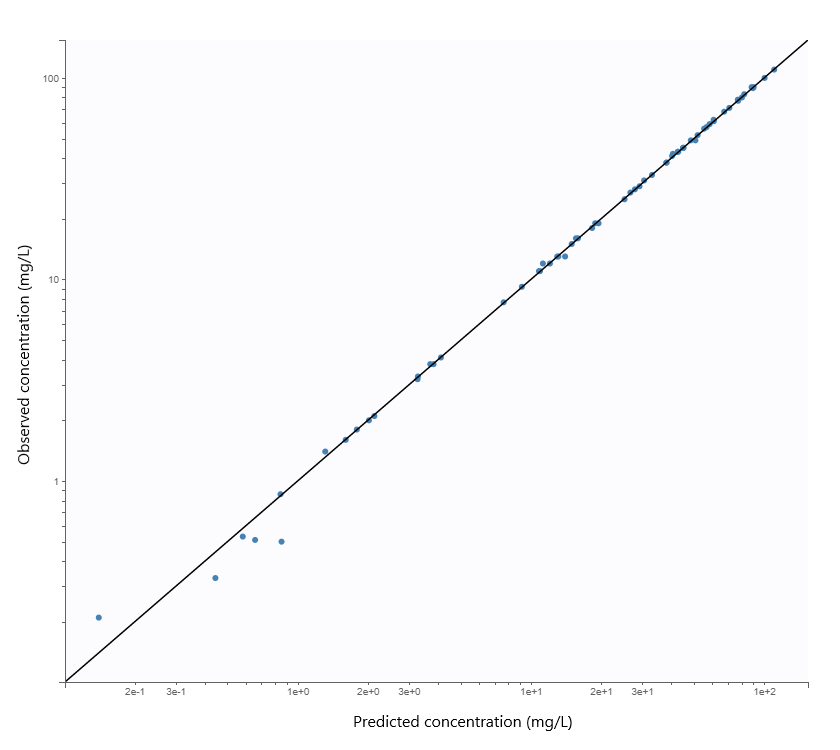
Figure S2C. Individual observations vs model predictions (*Li et al.* model, MER)

***Pharmacokinetic modelling***

The models were implemented in Monolix by fixing the initial values of population distribution parameters and variability measures in accordance with the values reported in the original publications^1,2^ and subsequently setting all SAEM iterations to zero, to keep initial estimates. Reported relative standard errors (RSE) were transformed to Monolix-preferred omega values when necessary, using the relationships: $\omega_{\psi}=\sqrt{\log\left( 1+\left( \frac{{SD}_{\psi}}{\psi} \right)^{2} \right)}$ and ${SD}_{\psi}=\frac{{RSE}_{\psi}}{100\%}\times\psi$ where ψ denotes the respective population pharmacokinetic parameter.

The mode of the conditional parameter distribution was subsequently estimated for every parameter and individual using the Metropolis-Hastings Markov chain Monte Carlo algorithm implemented in Monolix. These mode values, which correspond to the empirical Bayes estimates (EBE), were considered the most probable parameter values for each individual in our study given the observed concentrations and were used to predict concentration-time profiles for every patient (Supplementary Appendix, Figures S1-S2).

Individual estimates of achieved %*f*T>MIC were obtained in Simulx v.2021R2 (Lixoft©, Antony, Fr), by integrating a differential equation that tracked whether the predicted plasma concentration at each time point for every individual was above the MIC or not. To this end, a binary indicator variable ‘x’ was defined as ‘1’ when the predicted concentration exceeded the MIC and ‘0’ otherwise. The cumulative T>MIC was obtained by integrating the indicator over the duration of the dosing interval and 24h, respectively.

***Half-life calculations***

Terminal half-life (t½_β_) calculations were performed after converting Monolix-derived micro-rate constants (k10, k12, k21) to a macro-rate constant (β) for every patient and using the relationships^3^:

$k_{10}=\frac{CL}{V_{1}}$ (1)

$k_{12}= \frac{Q}{V_{1}}$ (2)

$k_{21}=\frac{Q}{V_{2}}$ (3)

where CL=clearance, Q=intercompartmental clearance, V_1_=central volume of distribution, and V_2_=peripheral volume of distribution. The mode of the conditional distribution for every parameter (empirical Bayes estimate) was used for the calculations of micro-rate constants. These micro-rate constants were subsequently converted to the macro-rate constant β using the relationship^3^:

β = $\frac{1}{2}(\left( k_{10}+ k_{12}+k_{21} \right)-\sqrt{\left( k_{10}+ k_{12}+ k_{21} \right)^{2}-4(k_{10}k_{21}}))$ (4)

Finally, the terminal half-life (t½_β_) was calculated for every patient according to the equation:

t½_β_ = ln(2)/β. (5)


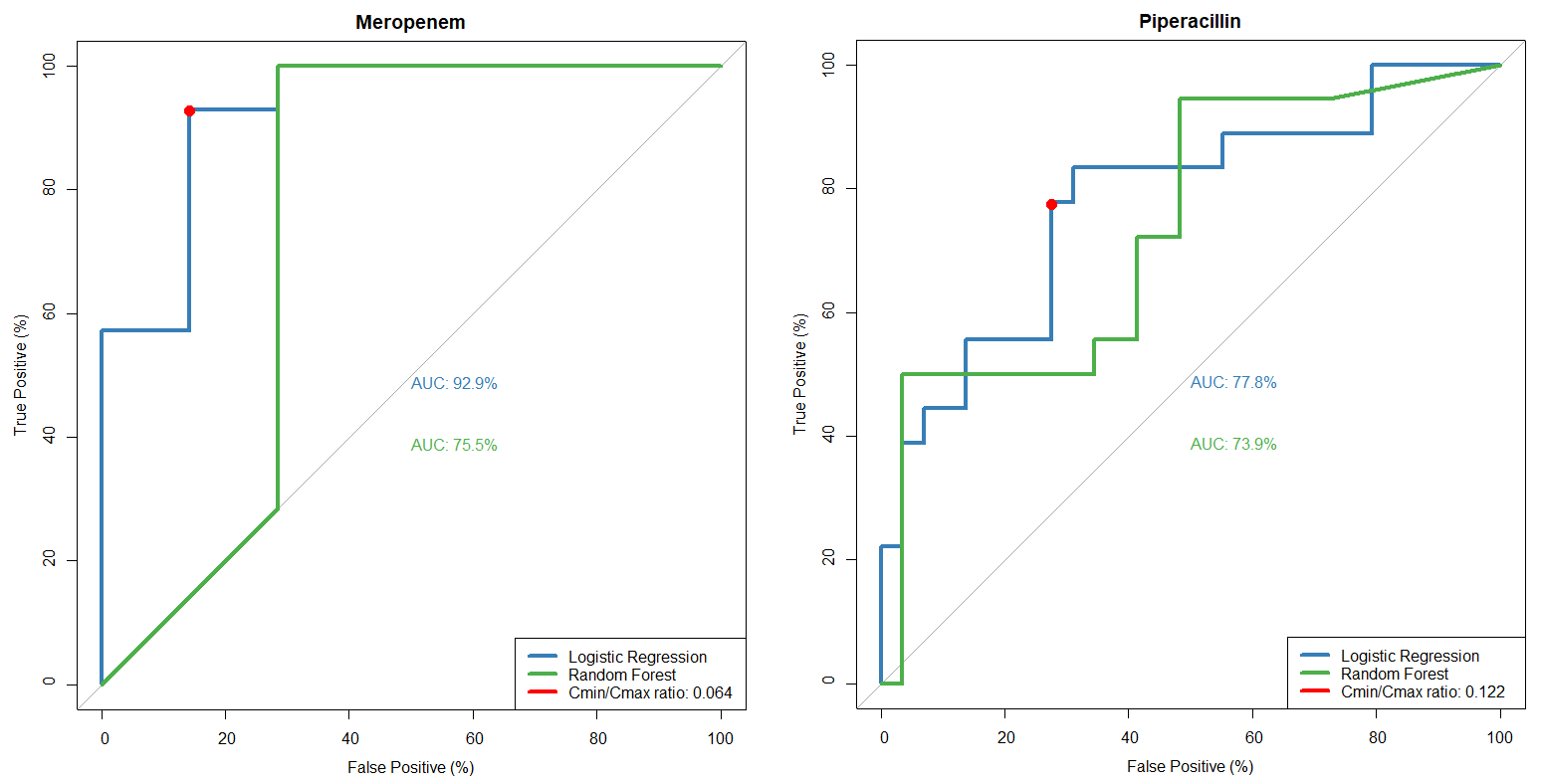
Figure S3. Receiver operating characteristic (ROC) curves for Cmin/Cmax ratio vs. ARC

ROC curves demonstrating the relationship between sensitivity and specificity for different values of the Cmin/Cmax ratio to discriminate between patients with and without suspected augmented renal clearance (ARC). The red dots indicate ideal cut-off values of the Cmin/Cmax ratio for the two drugs that provide the best trade-off between sensitivity and specificity.


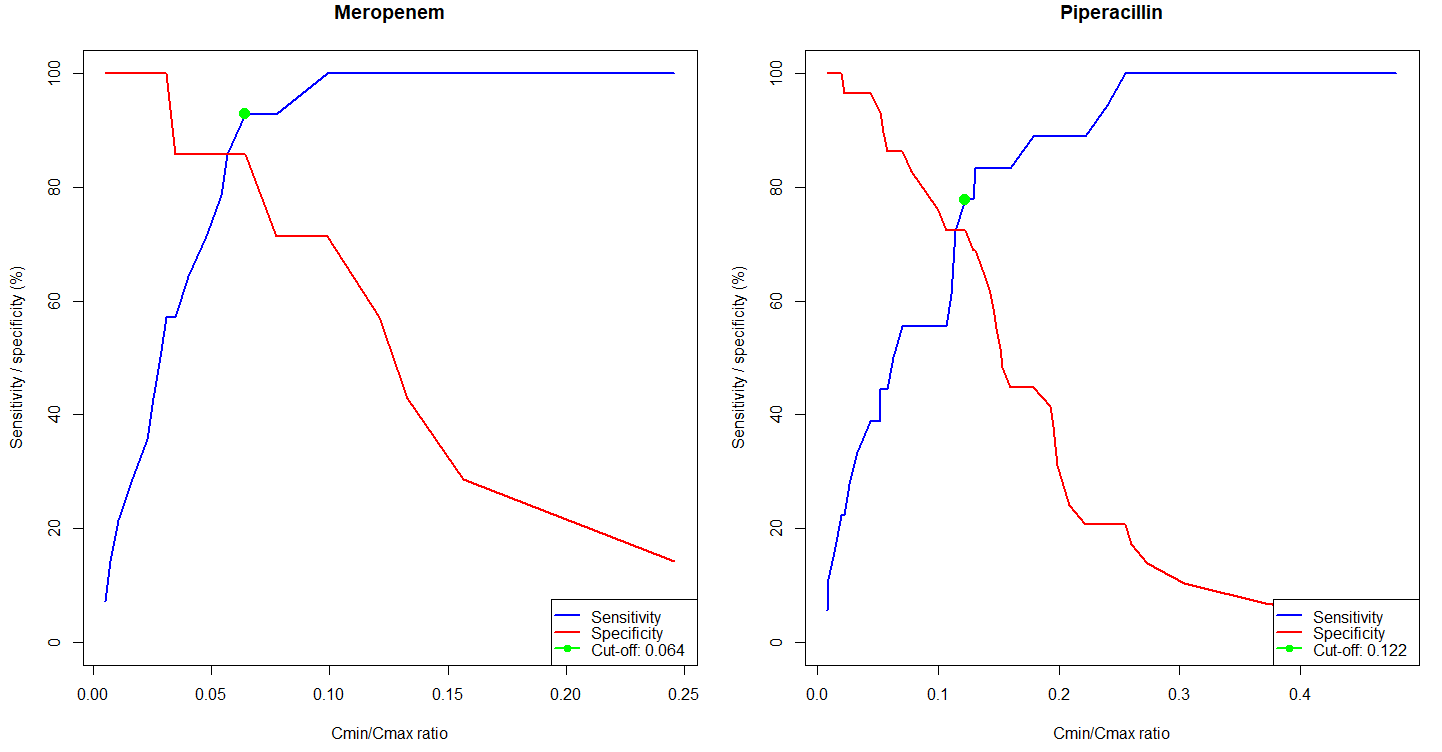
Figure S4. Cumulative distribution analysis (CDA) plots for Cmin/Cmax ratio vs. ARC

CDA plots visualizing the relationship between sensitivity and specificity for different values of the Cmin/Cmax ratio to discriminate between patients with and without augmented renal clearance (ARC). The green dots mark the optimal cut-offs where sensitivity and specificity are maximized. The cut-off for the meropenem Cmin/Cmax ratio of approximately 0.06 is thus associated with a 93% sensitivity and an 86% specificity to discriminate between patients with and without ARC. The corresponding sensitivity and specificity for piperacillin (at a cut-off Cmin/Cmax ratio of 0.12) are 77% and 72%, respectively.

References

1. Li C, Kuti JL, Nightingale CH, Nicolau DP. Population pharmacokinetic analysis and dosing regimen optimization of meropenem in adult patients. J Clin Pharmacol. 2006;46(10):1171-8.

2. Fillâtre P, Lemaitre F, Nesseler N, Schmidt M, Besset S, Launey Y, et al. Impact of extracorporeal membrane oxygenation (ECMO) support on piperacillin exposure in septic patients: a case-control study. J Antimicrob Chemother. 2021;76(5):1242-9.

3. Gabrielsson J & Weiner D. Pharmacokinetic and Pharmacodynamic Data Analysis - Concepts and applications. 5^th^ Ed. Swedish Pharmaceutical Society 2016.
